# PHC: the future of oncology drug development

**DOI:** 10.1186/1479-5876-10-S2-A12

**Published:** 2012-10-17

**Authors:** Yihong Yao

**Affiliations:** 1 Translational Sciences, MedImmune, LLC, Gaithersburg, MD, 20878, USA

## Background

Major investments in basic science have created an opportunity for significant progress in clinical medicine. Molecularly targeted therapies aim to interfere with molecular mechanisms, selectively involved in carcinogenesis and tumor growth in order to optimize the efficacy and minimize the side effects of anticancer treatment. Moving from concept to clinical use requires basic, translational, and regulatory science. Today, about 10% of labels for FDA-approved drugs contain pharmacogenomic information — a substantial increase since the 1990s but hardly the limit of the possibilities for this aspect of personalized medicine.

## Materials and methods

There has been an explosion in the number of promising markers but significant gap exists in independent analysis of the validity of the tests used to identify them in biologic specimens. The success of personalized medicine depends on having accurate diagnostic tests that identify patients who can benefit from targeted therapies.

## Results

In this presentation, case examples of how MedImmune/Astrazeneca incorporate personalized medicine/molecular diagnostic into clinical development to ensure targeted therapeutics successfully reach the right patient population will be discussed. These examples will illustrate how close cooperation among basic science, translational, clinical and regulatory departments together with partnerships with diagnostic companies are needed to reduce the dream of personalized healthcare into practice.

## Conclusions

By identifying the right patient population at the outset of the clinical development process we hope to improve the probability of success of oncology clinical trials, which is an urgent requirement across the industry, but especially for cancer patients with significant unmet need.

